# New Findings on the Crystal Polymorphism of Imepitoin

**DOI:** 10.3390/molecules29081724

**Published:** 2024-04-11

**Authors:** Giovanna Bruni, Doretta Capsoni, Anna Pellegrini, Angela Altomare, Mauro Coduri, Chiara Ferrara, Pietro Galinetto, Renato Molteni

**Affiliations:** 1 Department of Chemistry, Physical Chemistry Section & C.S.G.I. (Consorzio Interuniversitario per lo Sviluppo dei Sistemi a Grande Interfase), University of Pavia, 27100 Pavia, Italy; doretta.capsoni@unipv.it (D.C.); anna.pellegrini01@universitadipavia.it (A.P.); mauro.coduri@unipv.it (M.C.); 2 Institute of Crystallography—CNR, Via Amendola 122/o, 70126 Bari, Italy; angela.altomare@ic.cnr.it; 3 Department of Materials Science, University of Milano-Bicocca, Via Cozzi 55, 20125 Milano, Italy; chiara.ferrara@unimib.it; 4 Department of Physics, University of Pavia, Via Bassi 6, 27100 Pavia, Italy; pietro.galinetto@unipv.it; 5 A.M.S.A. Anonima Materie Sintetiche Affini S.p.A., Viale Giuseppe Di Vittorio 6, 22100 Como, Italy; r.molteni@amsacomo.it

**Keywords:** imepitoin, differential scanning calorimetry, polymorphism, monotropy, X-ray diffraction

## Abstract

Scientific and industrial reasons dictate the study of the solid state of imepitoin, a highly safe and tolerable anticonvulsant drug used in the therapy of epileptic dogs that was approved in the Europe Union in 2013. Our investigations allowed us to discover the existence of a new polymorph of imepitoin, which finds itself in a monotropic relationship with the crystalline form (polymorph I) already known and present on the market. This form (polymorph II), obtained by crystallization from xylene, remains metastable under ambient conditions for at least 1 year. Both solid forms were characterized by thermal (DSC and TGA), spectroscopic (FT-IR and Raman), microscopic (SEM and HSM), and diffractometric techniques. The thermodynamic relationship between the two polymorphs (monotropic) is such that it is not possible to study the melting of polymorph II, not even by adopting appropriate experimental strategies. Our measurements highlighted that the melting peak of imepitoin actually also includes an onset of melt decomposition. The ab initio structure solution, obtained from synchrotron X-ray powder diffraction data collected at room temperature, allowed us to determine the crystal structure of the new polymorph (II). It crystallizes in the monoclinic crystal structure, P2_1_/*c* space group (#14), with *a* = 14.8687(6) Å, *b* = 7.2434(2) Å, *c* = 12.5592(4) Å, *β* = 107.5586(8)°, V = 1289.61(8) Å3, and Z = 4.

## 1. Introduction

The crystalline polymorphism, resulting from different packing arrangements and/or molecular conformations of the same molecular entity in the crystal lattice, despite being a phenomenon known for over a century, still presents several obscure aspects today [1]. Indeed, it has not yet been possible to understand how to predict the number of different polymorphic forms in which a compound can exist, what the conditions and experimental procedures are that allow us to obtain them, and whether the thermodynamically stable crystalline form at ambient T and P has been ultimately obtained [2,3,4,5,6,7,8]. Essentially, it is not yet possible to achieve full control over the polymorph screening so much so that new solid forms are often obtained by chance rather than as the result of a systematic research.

Sometimes it is difficult to reproduce the formation of a certain polymorph even when the same experimental procedure that had previously produced it is followed to the letter. Therefore, even today, we must recognize the validity of the famous phrase of McCrone formulated in 1965: “every compound has different polymorphic forms, and, in general, the number of forms known for a given compound is proportional to the time and money spent in research on that compound” [9,10].

In the pharmaceutical field [11], these difficulties constitute a barrier to the physico-chemical and pharmaceutical characterizations essential for the development of the active ingredients from different points of view. In-depth knowledge of the polymorphic behavior of an active ingredient, indeed, is essential for choosing the most suitable crystalline form for the formulation, avoiding unpleasant inconveniences due to the appearance of new crystalline forms in advanced stages of the development of a drug, guaranteeing its maintenance during manufacturing and storage, and defending the patent [12,13,14,15,16,17,18].

**Scheme 1:**
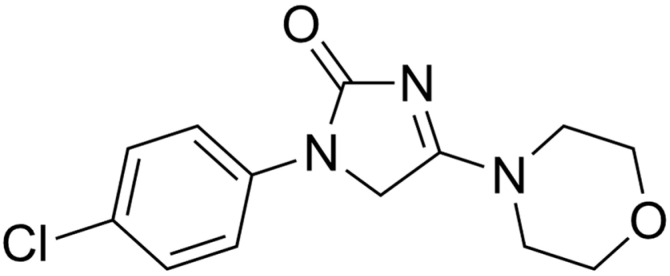
Molecular structure of imepitoin.

Based on these considerations, the study of the crystalline polymorphism of the active ingredients is even more important than ever, and the topic of this work arises precisely from the industrial need to delve deeper into the physico-chemical characterization and polymorphic behavior of imepitoin (Scheme 1), 3-(4-chlorophenyl)-5-morpholin-4-yl-4H-imidazol-2-one [19].

Imepitoin was developed in the 1990s from a series of imidazolinones. It has anxiolytic effects and a broad-spectrum anticonvulsant activity [20,21].

Imepitoin partially activates the receptors for the neurotransmitter GABA, a substance that reduces the electrical activity in the brain. In such a way, imepitoin increases GABA effects and helps to prevent convulsions, which in epilepsy, are due to abnormal electrical activity. It is the first compound with such a mechanism that has been developed as an antiepileptic drug and in dogs, shows several advantages over full agonists, such as less severe adverse effects and no dependence [22]. Based on randomized controlled trials that the demonstrated antiepileptic efficacy, high tolerability, and safety in epileptic dogs, the drug was approved for this indication in the Europe Union in 2013 [23].

Imepitoin is the active principle of Pexion^®^, available as tablets of 100 and 400 mg. The dose is calculated according to the dog’s weight (10–30 mg per kg bodyweight twice a day).

The crystalline structure of the only known solid form has been recently solved by Kadul [24]. This polymorph crystallizes in the *Pbca* space group with *a* = 12.35541(2) Å, *b* = 28.43308(8) Å, *c* = 7.340917(7) Å, and V = 2578.882(7) Å^3^ at 295 K. The roughly planar molecules stack along the *c*-axis. There are no conventional hydrogen bonds in the structure, but several intramolecular and intermolecular C–H⋯O, C–H⋯N, and C–H⋯Cl hydrogen bonds contribute to the crystal energy.

The only information present in the scientific literature regarding the physico-chemical behavior of this molecule is limited to the article cited above.

Thus, the aim of the present work was to study the thermal behavior of imepitoin, to investigate the possibility to obtain new polymorphs, and to comprehensively characterize the solids obtained using a wide range of analytical techniques: differential scanning calorimetry (DSC), thermogravimetry (TGA), hot-stage microscopy (HSM), powder X-ray diffraction (PXRD), scanning electron microscopy (SEM), FT-IR spectroscopy, Raman spectroscopy, and solid-state NMR (ssNMR) [25]. Obtaining this information is dictated by scientific interest and industrial needs.

New solid phases were searched for using several approaches [26]: grinding [27,28], sonication [29], quenching [30], kneading, and solvent crystallization.

Regarding the solvent crystallization, it is well known that the polymorphic outcome is often solvent-dependent since with changing the solvent, the solubility of the polymorphs and the interactions between the solvent and the solute molecules change, affecting the crystallization driving force and the nucleation and growth dynamics of the crystals [31].

Thus, several solvents were selected so as to include both a set of polar protic solvents (methanol, ethanol, isopropanol) of increasing molecular size as well as polar aprotic solvents (acetone, DMSO, DMF, and xylene) in order to expand the possibilities of obtaining new solid phases [32].

## 2. Results and Discussion

The sample of imepitoin as received will be indicated in the following as *ImeTQ*.

The *ImeTQ* powder was subjected to micronization (the micronized sample will be named *ImeGR* in the following), which is an important stage of the drugs manufacturing and, representing a step at risk of polymorphic transformation, is viewed with great apprehension by the pharmaceutical researcher.

*ImeTQ* was also crystallized from various solvents, including xylene. Only the crystallization from xylene leads to obtaining a solid with characteristics different from the original one. It will be named *ImeXyl* in the following.

To obtain new solid forms, in addition to the crystallization procedures, techniques such as sonication, quenching, and kneading were also used, but the products obtained did not bring to new solid phases, and these are not discussed in the following.

Details on the experimental procedures and characterization techniques are reported in Section 3.

### 2.1. SEM Measurements

**Figure 1:**
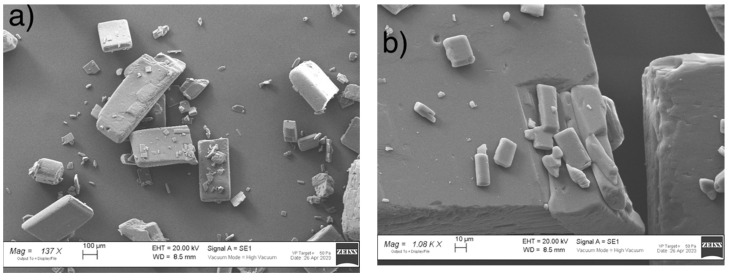

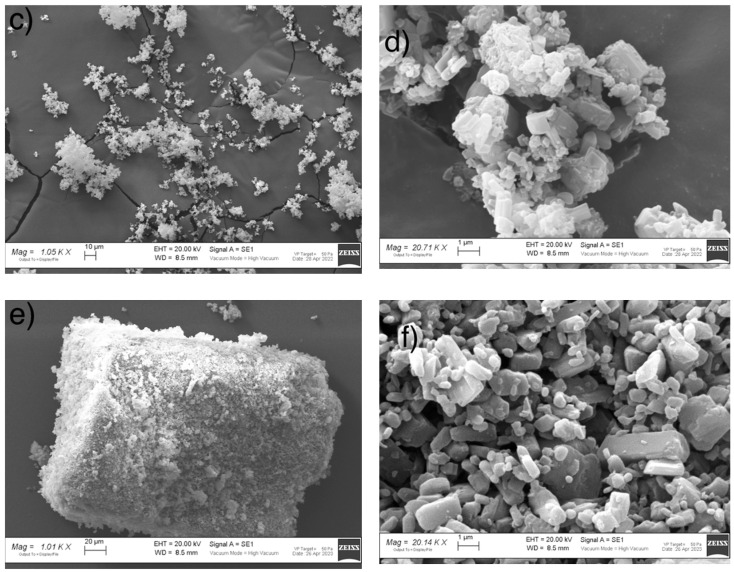
SEM photographs of *ImeTQ* (**a**,**b**), *ImeGR* (**c**,**d**), and *ImeXyl* (**e**,**f**).

Figure 1a,b show the SEM photographs collected of *ImeTQ*. It is possible to observe that the powder was made up of platelets with rectangular faces and very different sizes, ranging from a few microns to several hundred microns of length (Figure 1a,b). The surface of the particles was quite smooth.

After grinding (*ImeGR*), the appearance of the sample was completely different (Figure 1c,d). The particles assumed micrometric or even sub-micrometric dimensions, however, were much more homogeneous and had the shape of rods and platelets, often aggregated together.

The sample obtained by crystallization from xylene (*ImeXyl*) was made up of blocks of a few tens of microns covered by particles with size of a few micrometers and less than a micron (Figure 1e,f).

### 2.2. Thermal Behavior

#### 2.2.1. ImeTQ Sample

**Figure 2:**
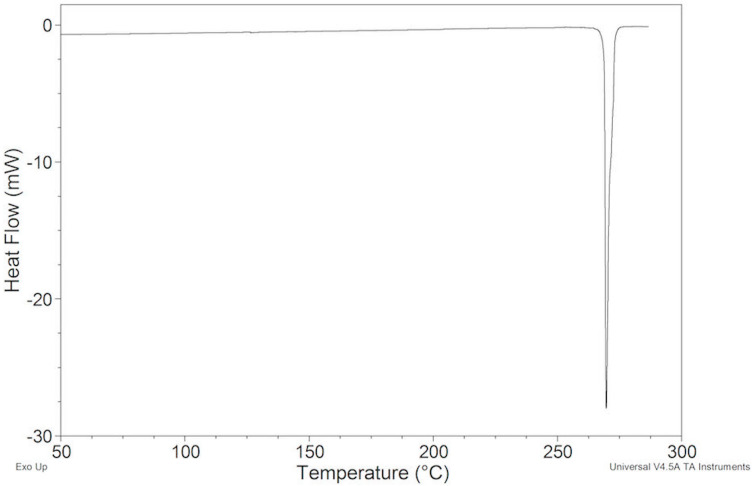
DSC curve of *ImeTQ* at β = 10 K min^−1^.

**Figure 3:**
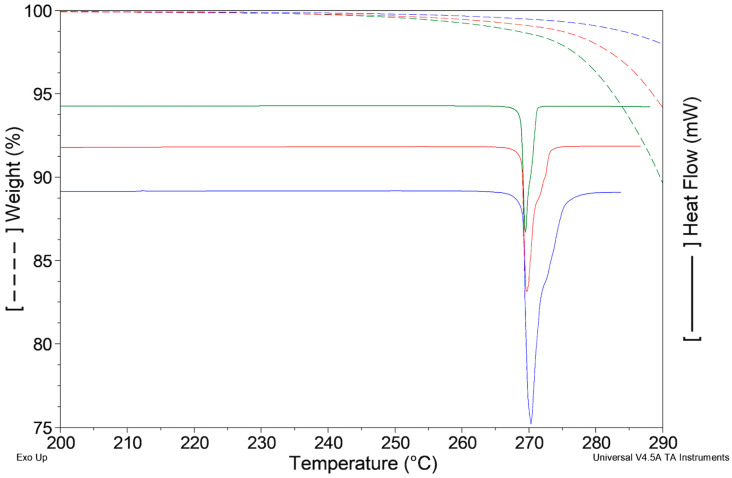
DSC (solid) and TG (short dash) curves of *ImeTQ* at three different β values: 5 (green), 10 (red), and 20 K min^−1^ (blue).

The DSC curve of *ImeTQ* presented, at all the heating rates investigated, a single endothermic peak, which was somewhat asymmetrical and which began very quickly. In Figure 2, it can be observed, as an example, the DSC trace of *ImeTQ* was heated at 10 K min^−1^, while in Figure 3, the DSC curves, together with the TG curves, performed at three different heating rates (β) (5, 10, and 20 K min^−1^), are shown in a graph with the X-axis expanded (200–290 °C) to provide a better view of the thermal effects. Indeed, the expanded temperature scale allows us to clearly observe how the DSC peak always began at the same temperature regardless of β and then ended at increasing temperature as β increased.

The onset temperature (*T*_onset_) was constant and equal to 268.97 ± 0.13 °C. The enthalpy change (Δ*H*) associated with the peak was also constant regardless of β and was 123.8 ± 0.8 J g^−1^.

The quantitative aspects of this behavior are compatible with a pure melting process, but the thermogravimetric analysis indicated that the sample began to lose mass significantly at high temperatures compatible with a decomposition process (Figure 3).

Considering that the sample heated up to 275 °C took on a yellowish color and when heated to a higher temperature, increasingly took on shades of brown, we are led to consider that a decomposition of the melt took place rather than its evaporation.

When β = 5 K min^−1^, the mass change measured at the closure of the DSC peak (273.1 °C) was 1.7%, with β = 10 K min^−1^ (275.2 °C) Δ*m* = 1.2% and β = 20 K min^−1^ (280.0 °C) Δ*m* = 0.9%. These values must be taken with due caution since the thermogravimetric and calorimetric signals were recorded with two independent instruments, and the experimental conditions in the two furnaces were certainly not identical. However, they indicate that the DSC peak was not due to a pure melting event—as β increased, the mass loss was lower. This trend can be explained by the fact that with increasing β, the thermal range is greater, but the duration of the melting process is shorter, and it is therefore compatible with the idea that the mass change due to decomposition of the melt is smaller. Indeed, at higher β the sample remained at high temperature for less time, slowing down the rate of progress of the decomposition.

The DSC peak is therefore essentially attributable to a melting process followed by decomposition of the melt. However, melt decomposition was very limited within the thermal range of melting, especially at 20 K min^−1^, and proceeded more quickly after the end of the melting peak.

**Figure 4:**
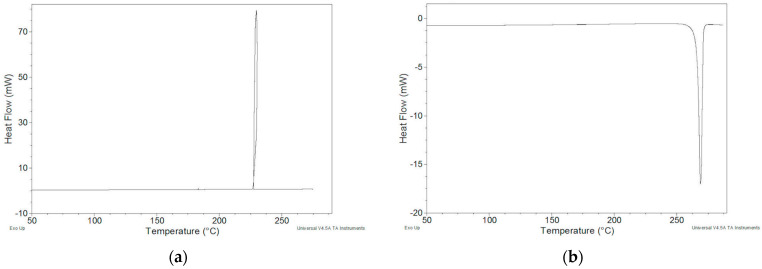
DSC curves of *ImeTQ*: (**a**) during cooling following a first heating to 275 °C and (**b**) during second heating at 10 K min^−1^.

To verify the reversibility of the endothermic peak, cyclic DSC measurements were carried out in an open pan which provided, for an initial heating at 10 K min^−1^, up to 275 °C (temperature just above the closing temperature of the DSC peak), a cooling at 2 K min^−1^ down to 30 °C, and a second heating at 10 K min^−1^ up to 290 °C. During cooling, an exothermic peak was recorded which we attribute to crystallization (Figure 4a). Indeed, in the second heating (Figure 4b), the endothermic peak was again present at the same *T*_onset_ value and was accompanied by a Δ*H* of about 2% lower than that obtained in the first scan (124.2 J g^−1^): 121.8 J g^−1^. The peak is therefore mostly reversible. Based on the thermogravimetric behavior of the sample, which suggests a certain decomposition of the melt, the decrease in Δ*H* in the second scan is predictable.

#### 2.2.2. ImeGR

The shape of the DSC curve of *ImeGR* did not differ from that of the unground sample (*ImeTQ*) (Figure S1). The quantitative data also were in excellent agreement with those obtained for *ImeTQ* (*T*_onset_ = 268.93 ± 0.13 °C, Δ*H* = 123.8 ± 0.8 J g^−1^). In fact, the *T*_onset_ was 268.82 ± 0.04 °C, and the ΔH was 124.6 ± 0.5 J g^−1^. This indicates that grinding did not induce any modification of the thermal behavior of the solid.

#### 2.2.3. Samples Recrystallized by Different Solvents

The DSC curves of the solids obtained by crystallization from various solvents (except for *ImeXyl* sample) were identical to those of the sample as such and of the micronized one. The quantitative data were also in good agreement.

#### 2.2.4. Samples Crystallized from Xylene

**Figure 5:**
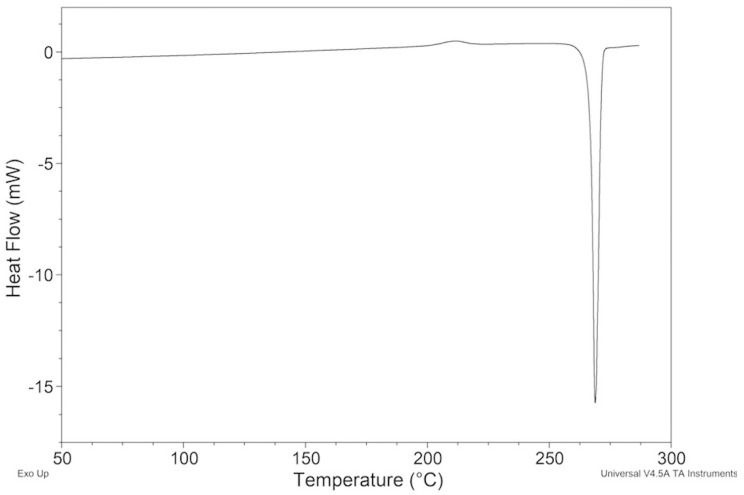
DSC curve of *ImeXyl* at 10 K min^−1^.

The thermal behavior of the samples crystallized from xylene (Figure 5) differed from that of all other crystallized samples due to the presence of a weak exothermic peak at *T*_onset_ = 202.16 ± 0.93 °C (10 min^−1^).

Its Δ*H* changed depending on the experimental conditions adopted in the crystallization. The highest value, 4.978 ± 0.119 J g^−1^, was obtained with the solid *ImeXyl* prepared by crystallization conducted under reflux with magnetic stirring and cooling of the solution to room temperature.

The exothermic peak was then followed by a melting peak that perfectly agreed with that shown by *ImeTQ* (Figure 5).

The reversibility of the exothermic peak was studied by performing a cyclic DSC measurement which involved an initial heating of up to 222 °C (i.e., until the exothermic peak ended) at 10 K min^−1^, a cooling down to 30 °C at 2 K min^−1^, and a second heating up to 290 °C at 10 K min^−1^. During the second heating, the exothermic peak was no longer visible while the melting peak was still present with the same thermal parameters of *ImeTQ*.

### 2.3. XRPD Measurements

#### 2.3.1. ImeTQ

**Figure 6:**
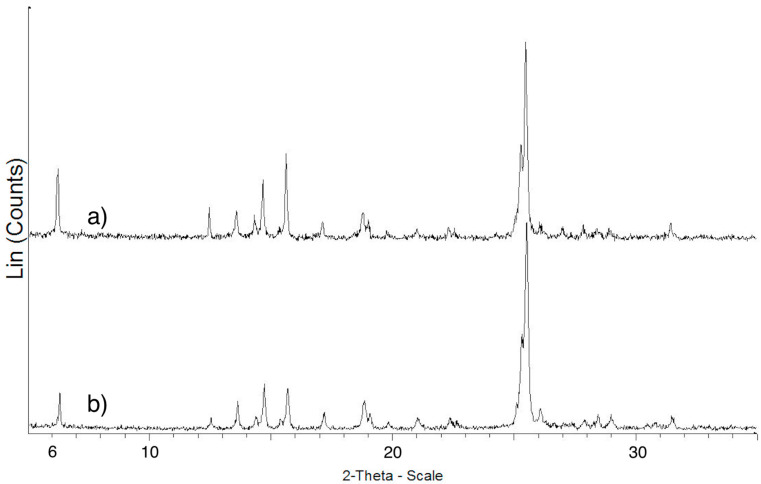
XRPD patterns of the samples *ImeTQ* (**a**) and *ImeTQ* heated up to 275 °C (**b**).

Figure 6 shows the XRPD pattern of *ImeTQ* compared with that of *ImeTQ* subjected to a cyclic measurement where the maximum temperature reached during heating was 275 °C (temperature corresponding to the DSC peak closure).

The patterns are both typical of crystalline powders and are practically identical. This is indicative of the fact that the decomposition of the melt is minimal and that the melting of imepitoin is an essentially reversible process as long as the temperature reached does not exceed the closing temperature of the DSC peak.

#### 2.3.2. ImeGR

As expected, based on the DSC results, the pattern of the *ImeGR* sample, except for the relative intensities of the peaks, was very similar to that of *ImeTQ* (Figure S2). All peaks were present in both patterns. The XRPD measurements therefore confirm the fact that grinding does not lead to changes in the solid form of imepitoin.

#### 2.3.3. Samples Crystallized from Various Solvents

As already seen for the thermal behavior, the diffractometric behavior of the samples prepared through the various crystallization procedures was also very similar. This rules out the possibility that the different crystallization procedures could lead to the formation of new polymorphs.

#### 2.3.4. ImeXyl

**Figure 7:**
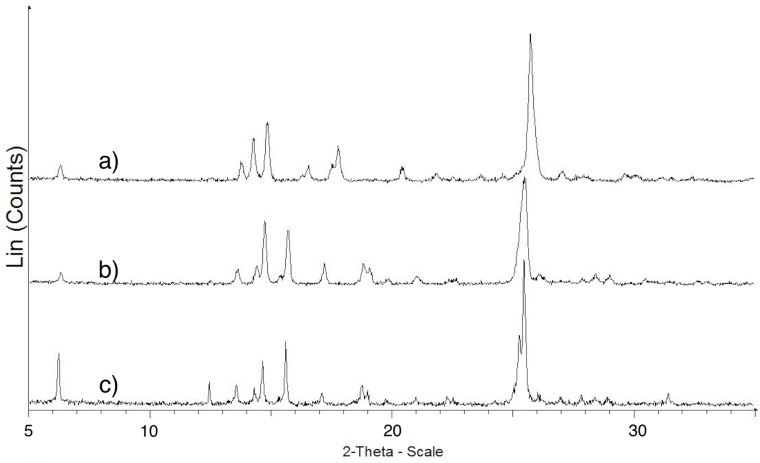
XRPD patterns of *ImeXyl* as is (**a**), *ImeXyl* heated to 230 °C, (**b**) and *ImeTQ* (**c**).

The XRPD pattern of *ImeXyl* differed substantially from all the others (Figure 7). Indeed, new peaks were present, and others were absent. In particular, the peaks at two-theta values 15.63, 17.12, 19.00, 20.95, and 25.39° were detected only for the ImeTQ and *ImeXyl* heated to 230 °C samples, and those at 16.49, 17.71, 20.35, 21.77, and 25.67° were characteristic of the *ImeXyl* one. This is evidence in favor of the fact that the sample was made up of a new crystalline form and allows us to rule out a crystallization process from an amorphous phase being the origin of the exothermic peak observed for this sample.

A decisive proof of the fact that the exothermic peak was due to a polymorphic transition comes from the comparison between the pattern of *ImeXyl* as such*, ImeXyl* heated in the DSC cell until the exothermic peak ended (230 °C), and the pattern of *ImeTQ* (see Figure 7).

As evident in the figure, the pattern of *ImeXyl* heated to 230 °C appears similar to that of *ImeTQ* and both differ from that of *ImeXyl* as is. This confirms the fact that during heating of *ImeXyl*, a polymorphic transition occurs, with the formation of the same polymorph of which *ImeTQ* is made.

This transition is not reversible.

The crystal structure of the new polymorph (II) was solved ab initio using synchrotron powder diffraction data. Accurate peaks positions were evaluated using standard peaks search methods, which was followed by profile fitting. The indexing procedure, based on the iterative use of singular value decomposition (SVD) algorithm [33] implemented in *TOPAS4.2*, gives a high quality primitive monoclinic cell (GoF(50) = 85.21), with *a* = 14.8703 Å, *b* = 7.2463 Å, *c* = 12.5652 Å, *β* = 107.555°, *V* = 1290.898 Å^3^, and Z = 4. The cell parameters identified by *TOPAS4.2* were also confirmed with the N-TREOR09 indexing program [34] implemented in EXPO software [35]. The *P*2_1_*/c* space group was identified via the EXPO automatic procedure based on the statistical–probabilistic analysis of systematic absences and validated via structure solution and refinement. The structure was solved using direct methods and also further confirmed using simulated annealing in EXPO.

**Figure 8:**
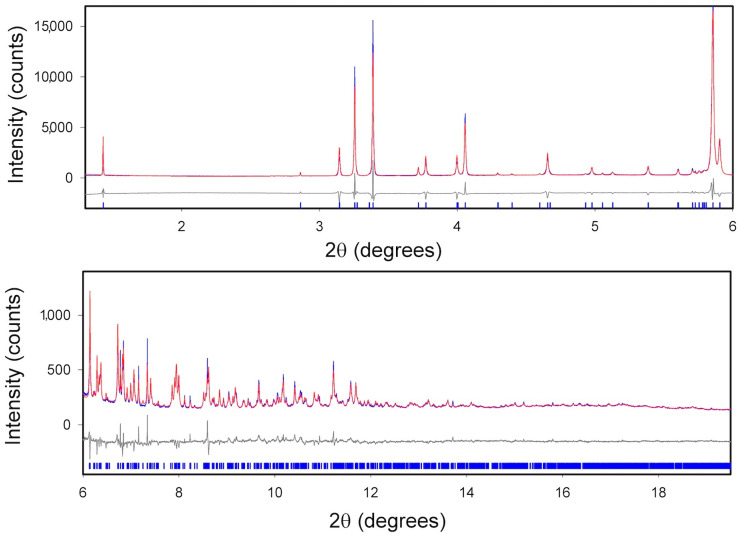
Rietveld refinement plot for the imepitoin monoclinic polymorph II: experimental (blue line) and calculated (red line) patterns, with difference plot (grey line) and peak markers (blue bars) at the bottom.

The Rietveld refinement [36] of the crystal structure was carried out in the 1.3–19.5° 2θ range. The polynomial function (Chebyshev type) was used to model the background, and the Pearson VII function was applied to refine the peak profile. Atomic displacement parameters were refined isotropically, and the overall atomic displacement parameters were refined for the groups of atoms (group 1: Cl, C5, C11, C6, C2, and C4 atoms; group 2: N1, C7, O1, N3, C3, and C8 atoms; group 3: C10, C11, N2, C12, C9, and O2 atoms). Hydrogen atoms bonded to carbon atoms were treated as riding under the constraint of atomic displacement parameters Uiso(H) = 1.2Uiso(C). Table S1 summarizes the X-ray crystallographic data collection and structure refinement for polymorph II. The final Rietveld refinement gives Rwp = 8.101, and the plot is shown in Figure 8. The CIF file is supplied as supporting information.

**Figure 9:**
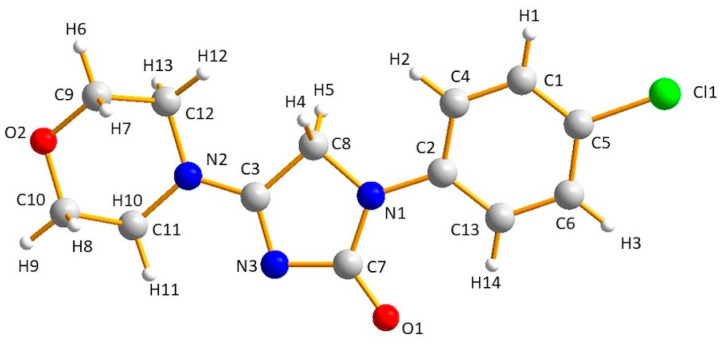
Imepitoin molecule with atom numbering.

The molecule with atom numbering is shown in Figure 9.

**Figure 10:**
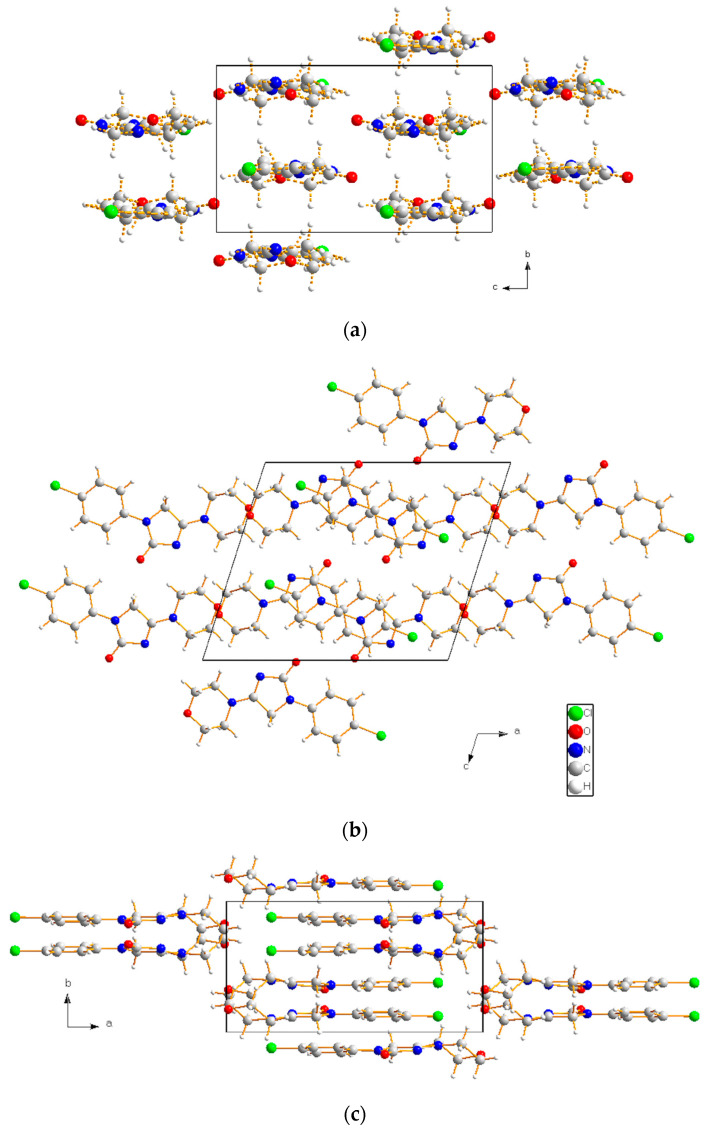
Imepitoin polymorph II crystal structure viewed down the (**a**) *a*-, (**b**) *b*-, and (**c**) *c*-axes.

Figure 10 shows the view of the packing of the imepitoin polymorph II down the a-, *b*-, and *c*-axes, generated using Diamond (version 3.0); the quite planar molecules are stacked along *b*.

Table 1 summarizes the possible C-H···O, C-H···N, and C-H···Cl intramolecular or intermolecular hydrogen bonds based on heavy atom-next neighboring hydrogen distances. In the same table, the corresponding distances for the known polymorph (polymorph I) are also reported; they were calculated based on the crystal structure described by Kaduk and coworkers [24]. The polymorph II displayed both stronger (O2···H6, O2···H10, and N3···H4) and weaker (O1···H14, O1···H4, N3···H12, Cl1···H10, and Cl1···H8) bonds than did polymorph I.

To complete the comparison of the new and the known polymorph, H···A and C···A distances discussed by Kaduk and coworkers for polymorph I [24] were also calculated for the new form. The distances are tabulated in Supplementary Materials (Table S2): overall the two polymorphs displayed comparable distances, whereas the H1···O1 and C1···O1 distances were significantly higher in polymorph II. The H1···O1 distances are highlighted in Figure S4.

The investigation of the torsion angles demonstrates that the imepitoin molecules are roughly planar in both polymorphs. The changes involve the C13-C2-N1-C7 and C4-C2-N1-C8 torsion angles (9.2 and 13.9° for polymorph II and −13.5 and −2.7 for polymorph I), suggesting that in polymorph, I the benzenic ring is slightly more tilted with respect to the imidazole ring as compared to polymorph II. This may explain the longer H1···O1 and C1···O1 distances detected in the new polymorph.

For a comparison of the crystal packing for the two polymorphs, the packing projection down each crystallographic direction for polymorph I is shown in Figure S5. There was a roughly planar molecule arrangement down the *b*- and *c*-axis for polymorph II (Figure 10) compared to that of polymorph I down the *c*- and *a*-axis. A different arrangement can be observed when comparing the view down the *a*- and *b*-axis for polymorph II and I, respectively. The planar imepitoin molecules result tilted in polymorph I.

### 2.4. Nature of the Polymorphic Transition of ImeXyl

The XRPD analysis confirmed the hypothesis that the exothermic peak measured in DSC for *ImeXyl* can be attributed to a polymorphic transition.

**Figure 11:**
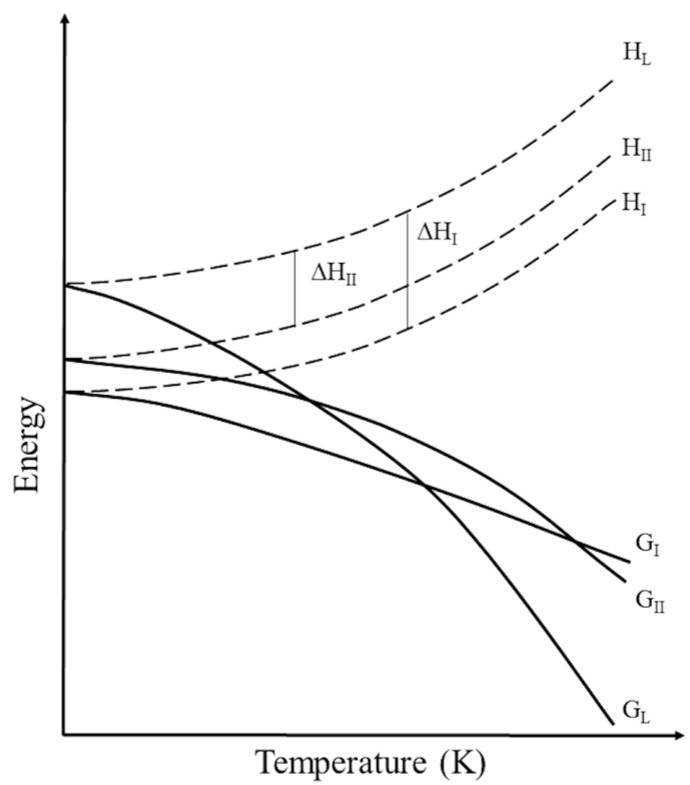
Energy-temperature diagram for a monotropic relationship.

According to the heat of transition rule proposed by Burger and Ramberger [37,38], if an endothermic peak is observed in the DSC curve, this indicates a transition between two forms that are related enantiotropically, while if an exothermic peak is observed, this indicates a transformation between two forms that are related monotropically (Figure 11).

Based on this rule, the exothermic nature of the peak suggests that the polymorphic relationship is monotropic, where the new polymorph, the one corresponding to *ImeXyl*, is the metastable polymorph, which we will call polymorph II, while that of the other samples analyzed is the polymorph stable at all *P* and *T* values. We will call it polymorph I.

We were unable to measure the *T*_onset_ and Δ*H* of the melting of metastable polymorph II, as it transformed into the stable polymorph before melting. According to Burger and Ramberger’s rules [37], polymorph II should melt at a lower temperature than should polymorph I and with a lower Δ*H*.

**Figure 12:**
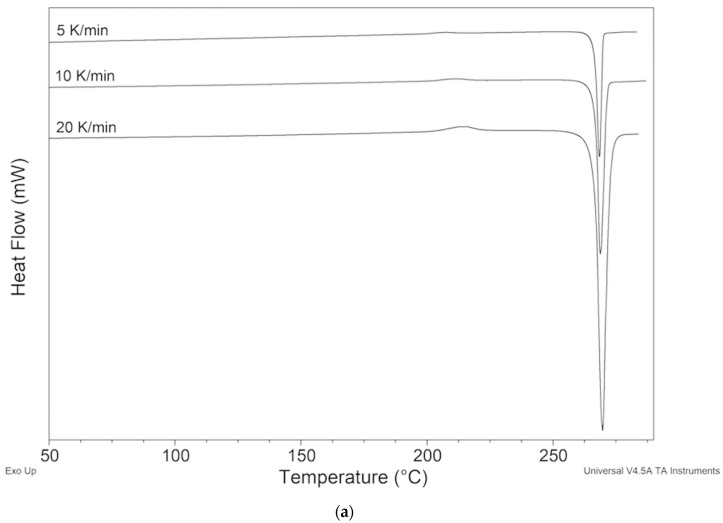

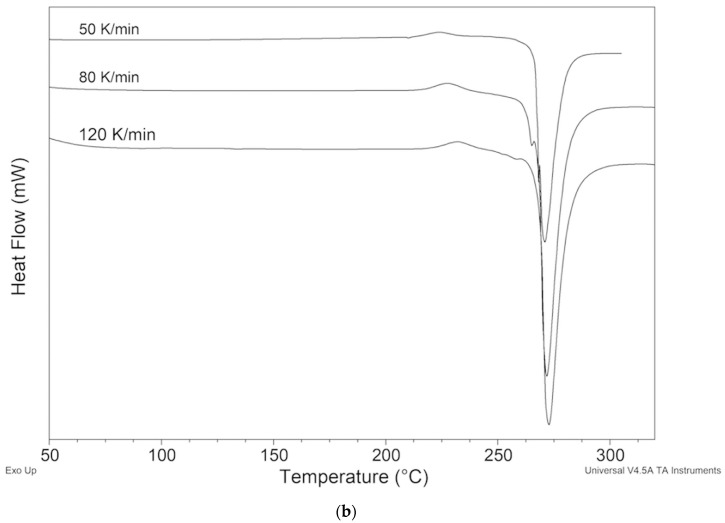
DSC curves of *ImeXyl* at different β values. (**a**) 5, 10 and 20 K min^−1^; (**b**) 50, 80, and 120 K min^−1^.

To prevent the polymorphic transition and to be able to measure the melting of polymorph II, we attempted to act on the kinetic factor by designing DSC measurements at a high heating rate, up to 120 K min^−1^ (Figure 12). The exothermic peak shifted to higher temperatures but was always present. This behavior made it impossible to experimentally measure the temperature and the melting enthalpy of polymorph II.

We could only obtain the Δ*H* value by considering its correspondence to the algebraic sum of the melting enthalpy of form I and the transition enthalpy. We obtained the value of 118.82 J g^−1^.

The melting temperature cannot even be obtained theoretically since when the polymorphs are in a monotropic relationship, the exothermic transition is kinetically driven and appears at a variable temperature. Thus, Yu’s equation [39] is not applicable.

### 2.5. Measurements HSM

#### ImeXyl

The visual observation of the polymorphic transition was conducted via HSM.

**Figure 13:**
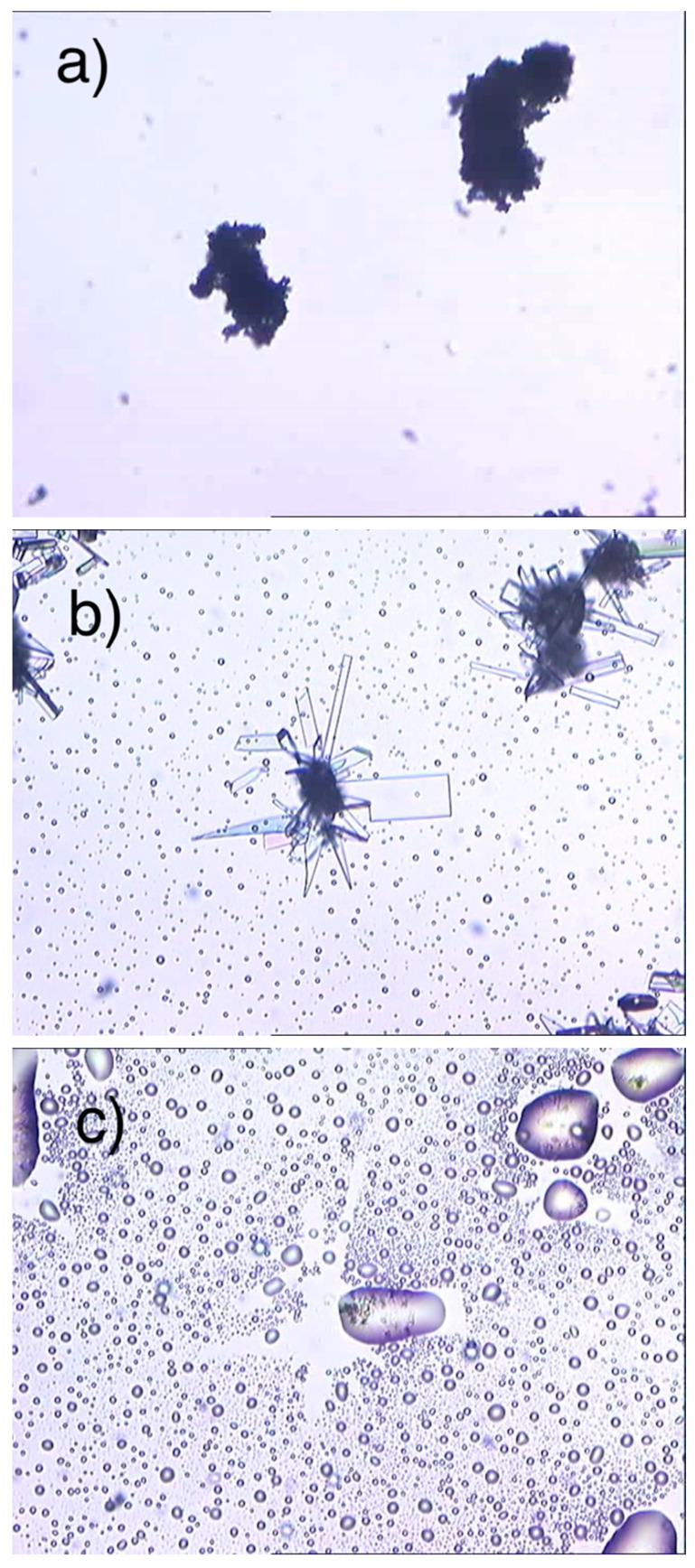
Photographs of *ImeXyl* at (**a**) 180 °C, (**b**) 230 °C, and (**c**) 275 °C (images taken at a magnification of 10×).

During the HSM analysis, the morphology of *ImeXyl* did not change (Figure 13a) until the temperature corresponding to the exothermic peak observed in DSC was reached. At that point, an important transformation of the particles was observed with the sudden formation of new crystals with well-defined geometric contours (Figure 13b). This was the solid phase (polymorph I) that formed from the starting polymorph (II). Once the *T* measured in DSC for the melting of the sample was reached, the liquefaction of the sample was observed with the formation of droplets of liquid (Figure 13c).

Therefore, the HSM analysis provides further confirmation of the fact that the exothermic peak is due to the polymorphic transition of the solid.

### 2.6. FT-IR Spectroscopy Measurements

#### 2.6.1. ImeTQ and ImeGR

**Figure 14:**
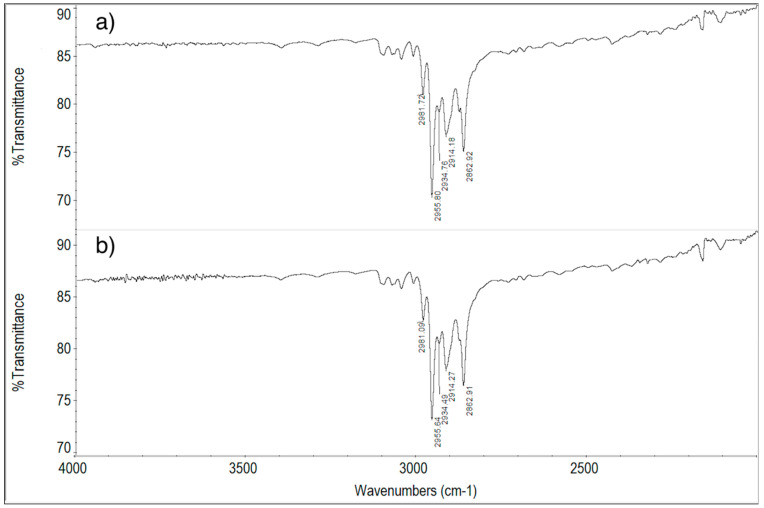

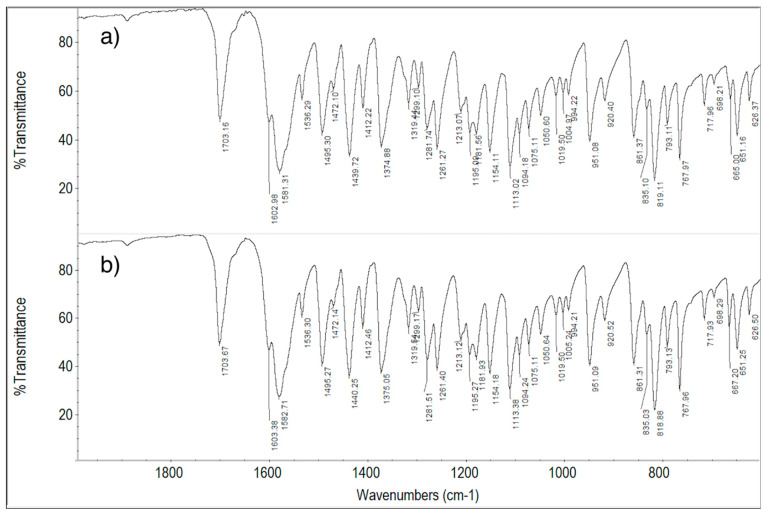
Spectra FT-IR of *ImeTQ* sample (**a**) and *ImeTQ* heated to 275 °C (**b**).

The attributions of peaks/bands of the FT-IR spectrum of *ImeTQ* sample (Figure 14) to the vibrations of the imepitoin molecule are listed in Table 2.

The spectrum of the *ImeTQ* sample is reported together with that of the same sample subjected to cyclic measurement with a maximum temperature reached during heating of 275 °C (temperature corresponding to the DSC peak closure) in Figure 14. The spectra were identical, confirming the fact that the decomposition of the newly formed melt is very minimal so much so that its products are not detectable in the FT-IR spectrum.

The FT-IR spectrum of the *ImeGR* sample was very similar to that of the unground sample, confirming the fact that grinding does not cause any changes in the imepitoin sample.

#### 2.6.2. ImeXyl

**Figure 15:**
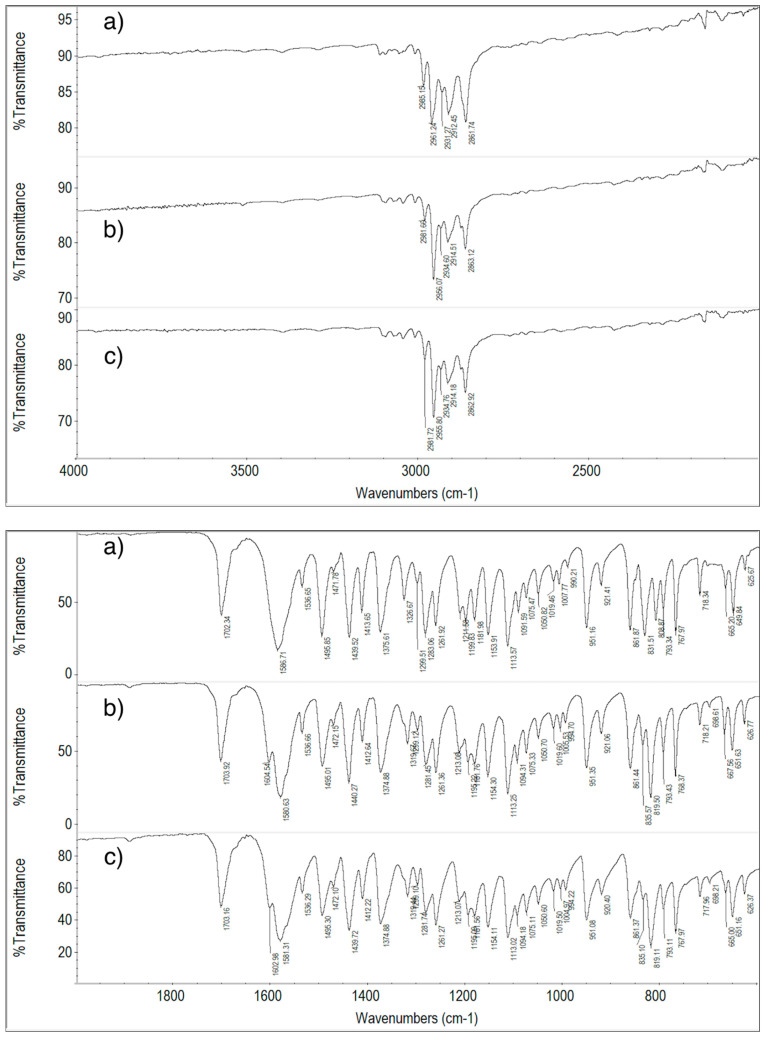
FT-IR spectra of *ImeXyl* (**a**), *ImeXyl* heated up to 230 °C (**b**), and *ImeTQ* (**c**).

In Figure 15, the FT-IR spectra of *ImeXyl, ImeXyl* heated to 230 °C and *ImeTQ* samples are shown.

The differences between the spectrum of the *ImeXyl* sample and the others were not particularly numerous, and they only concerned the absorptions around 1600, 1280, and 800 cm^−1^.

The FT-IR technique, therefore, provides weak but genuine evidence that the *ImeXyl* sample is made up of a solid phase different from that of *ImeTQ* sample. The light differences might have been due to the fact the vibrational scheme was dominated by molecular modes with a minor contribution from intermolecular bonds.

The FT-IR technique confirms the fact that when a *ImeXyl* sample is heated to 230 °C, it undergoes a transition to polymorph I. Indeed, the spectrum obtained after the heat treatment returned to the same as that of the *ImeTQ* sample.

### 2.7. Raman Measurements

Further confirmation regarding the existence of polymorphic imepitoin were derived from Raman results.

In Figure S3a, the room-temperature Raman spectra for *ImeTQ* and *ImeXyl* are reported in the range 100–3200 cm^−1^. As expected, in both cases, several Raman lines were well resolved. It is important to note that the reported spectra were obtained from the average of five different spectra acquired from different regions for each sample. In addition, all the measurements were obtained by focusing a red laser light to a 10x microscopic objective, leading to a sampling area of about 100 micron^2^.

Raman sampling of polymorphism in complex molecules involves the detailed analyses of the whole energy range due to the relevant effect on both lattice modes, typically effective in the low-energy region, approximately below 200 cm^−1^, and inter- and intra-molecular unit modes with Raman signatures extending to 3500 cm^−1^ [40].

The Raman signal of the imepitoin is the convolution of different Raman active units: the aromatic, imidazole, and morpholine rings; and several intramolecular and intermolecular C–H⋯O, C–H⋯N, and C–H⋯Cl hydrogen bonds. Likely, one can roughly classify the main intense Raman modes (i) in the range 200–700 cm^−1^ as due to C-C and C-Cl vibrations, (ii) in the range 800–2000 cm^−1^ as due to the different ring vibrations, and (iii) in the range 2800–3200 cm^−1^ as due to CH_n_. In the region (ii), the CC and CN stretching modes are active as well [41,42,43,44].

In the following, we limit our comments to those modes displaying Raman peak changes greater that 5 cm^−1^ and/or displaying clear changes in shape.

To better appreciate changes from *ImeTQ* and *ImeXyl*, in Figure S3b–d, the different regions are reported. From enlarging the scale, it is evident that different Raman features were shifted or changed.

For *ImeTQ* powder in the lower energy region, the doublet at around 560 cm^−1^, is probably associated with the C-Cl stretching mode having an energy separation of about 10 cm^−1^ with an asymmetric intensity distribution. On the contrary, for *ImeXyl* sample, the doublet appears more symmetric but with a greater energy gap, i.e., 14 cm^−1^. It is important to note that at higher energies, the main intense features at around 800 cm^−1^, probably due to ring deformation, remain unchanged.

In the region between 1000 and 1800 cm^−1^, the main Raman signals should be attributed to the vibrations inside the morpholine and imidazole ring, to the CN, and to CC stretching to eventually be coupled with C-H bending. The main features at around 1180, 1380, 1600, and 1700 cm^−1^ appeared to be substantially unchanged. Some spectral changes passing from *ImeQT* to *ImeXyl* could be otherwise observed at around 1320 cm^−1^ and 1450 cm^−1^. These two Raman complex structures (see arrows in Figure S3c) exhibited a clear splitting for *ImeXyl*, suggesting a different symmetry for the involved vibrational cages.

Finally, in Figure S3d, the region between 2800 and 3200 cm^−1^ is reported. At these energies, symmetric and antisymmetric stretching vibrations of CH_2_ and CH_3_ bonds were Raman active. Again, we observed clear differences moving from *ImeTQ* to *ImeXyl*: (i) a shift at higher energies of about 6 cm^−1^ for the mode at 2960 cm-^1^, (ii) the merging of the doublet around 2980 cm^−1^, and (iii) a marked increase in the energy separation between the two peaks at around 3070 cm^−1^. The observed Raman spectral differences between *ImeQT* and *ImeXyl* are consistent with a polymorphic behavior.

### 2.8. NMR Measurements

For *ImeTQ* and *ImeXyl*, the ^13^C NMR spectra were recorded in order to highlight the main differences in the local structure. In general, solid-state NMR is a powerful tool for the analysis of the polymorphs of organic and inorganic compounds, as it allows for the investigation of the local structure and therefore highlights the differences in short-range order, determining the groups involved in interactions and changes in local geometry.

**Figure 16:**
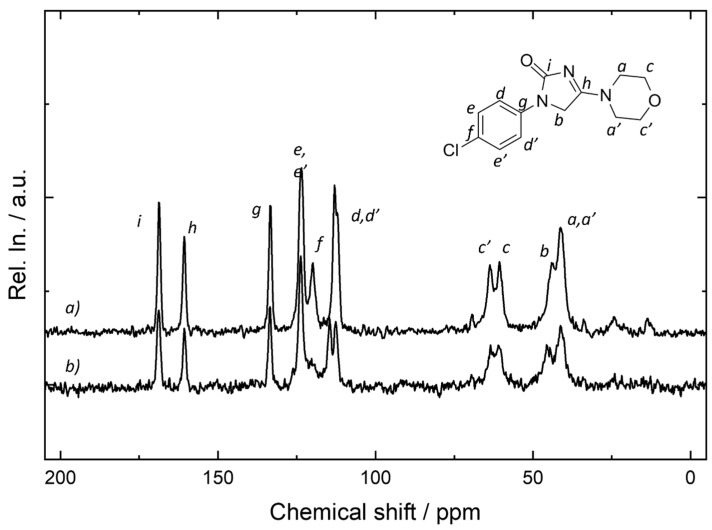
^13^C cpmas spectra (10 kHz) of *ImeTQ* (**a**), *ImeXyl* (**b**), and the corresponding attributions.

The ^13^C spectra of *ImeTQ* and *ImeXyl* are shown in Figure 16. All resonances can be attributed to the imepitoin sample since the presence of other species in the powders was not expected.

While the signals related to the imidazole and morpholine rings were essentially unchanged between the two forms, the main differences were found in the region of aromatic signals, in good agreement with the evidence from the FTIR analysis. In fact, in the region at 120–140 ppm, doubling of the d signal and the variation in the intensity of the f signal were observed, while no shift was observed for any of the peaks. The other signals underwent very limited variation in the relative intensity. As the two spectra were acquired with the same experimental parameters, the variation in the intensity can be correlated to changes into the sample and specifically to the efficiency of the cross-polarization effect at the base of the CPMAS technique. In this sense, the signals i,h and a, a’ show the main variation in relative intensity compared with the aromatic signals and by comparison between the two samples. As surmised from the attribution, these signals were related to the C=O and C-H position, thus further confirming the analysis from FTIR. It must be stressed that these variations are small, and thus subtle differences among the two species can be expected from the point of view of the local structure. Indeed, this scenario is compatible with a small change in the conformation of the molecule and with a set of intermolecular interactions (e.g., the interacting groups and the distances between the groups remaining essentially the same), probably driven by a tilt or rotation of a specific group. The splitting of the d signal into two resonances suggests a lowering of the symmetry of the crystalline structure.

### 2.9. Kinetic Stability of the Polymorph II

The polymorph obtained by crystallization of imepitoin powder from xylene maintained identical thermal, spectroscopic, and diffractometric behavior 12 months after its preparation under ambient conditions. This therefore indicates that polymorph II remains metastable for at least a period of 12 months.

## 3. Materials and Methods

### 3.1. Materials

The imepitoin powder (purity 98.0–102.0%) was kindly donated by the pharmaceutical company AMSA S.p.A. (Como, Italy).

### 3.2. Grinding

The powder *ImeGR* was obtained by micronization (working pressure 7 bar; counter-rotating screw: 400 rpm) in a ProMill8 device (FPS, Como, Italy).

### 3.3. Solvent Crystallization

The imepitoin powder as received was subjected to crystallization from several solvents: acetone, ethanol, n-propanol, methanol, DMSO, DMF, and xylene. Different experimental conditions were used: *ImeTQ* was suspended in each solvent and then heated (by varying the heating time and temperature) without or under reflux until the powder was completely dissolved. The solution obtained was then left to cool spontaneously to room temperature or cooled in water and ice. The crystals obtained were then filtered.

*ImeXyl* was obtained by suspending 0.5 g of *ImeTQ* in 300 mL of xylene, then heating to 130 °C under reflux for 3 h (with magnetic agitation), and finally spontaneously cooling to room temperature. Buckner filtration was used to filter the suspension obtained. The solid was dried for 12 h at 90 °C under vacuum.

### 3.4. Sonication, Quenching, and Kneading

Sonication was applied using a Falc mod. LBS1 (BG, Italy) instrument with a power of 100 W and a frequency of 40 kHz for 15 min.

Imepitoin was quenched by removing the DSC pan after melting of *ImeTQ* and placing it into liquid nitrogen.

*ImeTQ* was manually ground for 40 min in an agate mortar, with a few drops of solvent (ethanol, xylene) being added from time to time. The product so obtained was spontaneously dried at room temperature.

### 3.5. SEM Measurements

The samples were fixed with carbon tape on a stub and made conductive owing to the deposition on their surface of a small layer of gold in an argon atmosphere to make interactions among the electrons possible. Then, they were observed with a Zeiss mod. EVO MA10 scanning electron microscope (Carl Zeiss, Oberkochen, Germany) with an accelerating voltage of 20 kV at different magnifications.

### 3.6. Thermal Measurements

Calorimetric and thermogravimetric data were collected with a DSC Q2000 and a TGA Q5000 analyzer, both interfaced with a TA5000 processor (TA Instruments, New Castle, DE, USA). Calibration of temperature and enthalpy scales in the DSC apparatus was conducted by running ultrapure indium standard (purity, 99.999%; melting point = 156.6 °C; melting enthalpy, Δ*H* = 28.54 J g^−1^).

The analyses were performed in a flow of dry nitrogen (50 mL min^−1^) in an open aluminum crucible (DSC) or platinum holder (TG) on powder masses of approximately 3–4 mg at different heating rates. Furthermore, differently designed cyclic measurements were carried out.

All experimental thermal data are averages of at least three experiments. Typically, temperatures were reproduced within 1 °C.

### 3.7. XRPD Measurements

The diffractograms were collected with a Bruker D5005 diffractometer (Siemens, Karlsruhe, Germany) equipped with a vertical goniometer, curved graphite crystal monochromator, and a scintillator detector. Cu Kα radiation (λ = 1.54056 Å) was used. The measurements were performed at room temperature in the 2θ angular range between 5 and 35° (voltage = 40 kV; current intensity = 40 mA) in the step scanning mode (step width = 0.020°; counting time = 2 s step^−1^).

The high-resolution powder pattern of the new imepitoin polymorph was collected at room temperature (25 °C) at the ID22 high-resolution powder diffraction beamline of the European Synchrotron Radiation Facility (ESRF) [45] in transmission geometry using a setup equipped with crystal analyzers [46]. The radiation wavelength, calibrated against a silicon standard (NIST SRM 640c), was set to 0.35418 Å, which corresponded to ~35 keV. Beam size was set to 1.0 horizontal by 0.9 mm vertical. The powder was filled into a borosilicate capillary with a 1.0 mm diameter, which was mounted on the axis of the diffractometer and spun during acquisition to improve powder randomization.

In order to minimize radiation damage, the pattern was obtained by averaging many fast continuous scans (10 deg. min^−1^ scanning rate) recorded after translation of the capillary by 1.2 mm along its axis.

This approach minimized the exposure of the specimen to the radiation, thus exposing, for every fast acquisition, a fresh portion of the specimen.

The final pattern was obtained by integrating the diffraction intensity up to 36 deg. with a step of 0.002 deg. for a total acquisition time of 30 min.

The pattern was indexed using *TOPAS4.2* software [47]. The ab initio solution and the structure refinement process were automatically performed using EXPO software [35], a comprehensive package capable of carrying out all the following steps: determining unit-cell parameters and identifying the spacer group; b) solving the structure model using direct methods and/or a real-space approach based on simulated annealing; and d) refining the structure model using the Rietveld method [36]. The indexing process in EXPO validated the cell parameters identified by *TOPAS4.2*. The space-group determination was automatically executed based on the evaluation of the systematic absences. The structure was solved using direct methods and also confirmed through simulated annealing.

### 3.8. HSM Measurements

For HSM measurements, a Nikon microscope, the Eclipse ME600 (Nikon, Inc., Melville, NY, USA), equipped with a THMS600 heating oven with temperature control (Linkam Scientific Instruments Ltd., Surrey, UK) and a JVC video camera was used. Samples were placed on glass microscope slides with a cover plate and heated at 10 K min^−1^ until melting was completed.

### 3.9. FT-IR Measurements

The spectroscopic measurements were conducted using an iS20 FT-IR spectrometer (Nicolet, Madison, WI, USA) equipped with an accessory for attenuated total reflectance sampling (ATR; smart iTR with diamond crystal) working in the spectral range of 4000–600 cm^−1^.

A total of 32 scans of the moving mirror were collected to obtain a high signal-to-noise ratio. The resolution was 4 cm^−1^.

### 3.10. Raman Measurements

Raman measurements were performed at room temperature on the investigated samples using an automated and integrated confocal microRaman spectrometer, the XploRA Plus HORIBA Scientific (Osaka, Japan), equipped with an Olympus BX43 microscope. The microscope setup consisted of four different objectives with magnifications of 10×, 50×, 50× with a long working distance, and 100×. The spectrometer was equipped with a motorized xy stage on which the investigated samples were positioned. The spectral resolution was about 1 cm^−1^. An Open Electrode CCD camera with a multistage Peltier air-cooling system was used as the detector. A solid-state laser source was used to emit light at 638 nm. The maximum power was 90 mW.

### 3.11. NMR Measurements

The ^13^C cpmas spectra were acquired on a Bruker Avance III 9.4 magnet equipped with a 4 mm rotor under 10 kHz rotation conditions with a contact time of 2.5 ms, a delay time of 8 s (calibrated to the delay time of the proton), and 2k acquisitions, using a rotor with a spacer due to the small amount of the sample. The spectra were referenced to adamantane signals as a secondary standard.

## 4. Conclusions

A new polymorph (II) was found for imepitoin. It is in a monotropic relationship with the already-known crystalline form that is present on the market (polymorph I). Polymorph I is the stable phase at any value of T and P. Polymorph II that can be obtained by crystallization from xylene is never stable, but it holds as a metastable form under ambient conditions for at least 1 year.

The thermodynamic relationship between the two polymorphs (monotropic) is such that it is not possible to study the melting of polymorph II, not even by adopting appropriate experimental strategies. Our measurements indicated that the melting peak of imepitoin actually also includes an onset of melt decomposition.

The ab initio structural solution, obtained from synchrotron X-ray powder diffraction data collected at 25 °C, allowed us to determine the crystal structure of the new polymorph (II). It crystallizes in the monoclinic crystal structure, P21/*c* space group (#14), with *a* = 14.8687(6) Å, *b* = 7.2434(2) Å, *c* = 12.5592(4) Å, *β* = 107.5586(8)°, V = 1289.61(8) Å^3^, and Z = 4.

The present study provides the necessary basis for a conscious choice of the crystallization process of imepitoin and of the techniques for characterizing the powder obtained so as to avoid problems in the subsequent phases of the drug’s life.

## Figures and Tables

**Table 1:** A···H (A = O, N, Cl) distances (Å) in imepitoin polymorph II compared to those in polymorph I. For atom numbering, refer to Figure 9. In parenthesis, the hydrogen bonds displaying close distances are reported.

H-Bond	A···H (Å) (II)	A···H (Å) (I)
O2···H6 (or H7, H8, H9) O2···H8 (or H13) O2···H10	1.96–1.99 * 2.66–2.70 2.82	2.09–2.19 * 2.62 2.96
O1···H14 O1···H4 O1···H12	2.24 * 2.35 2.78	2.10 * 2.03 2.77
N3···H11 N3···H6 N3···H4 N3···H12	2.41 * 2.93 3.04 * 3.04	2.39 * 2.87 3.23 * 3.12
Cl1···H1 (or H3) Cl1···H10 Cl1···H8	2.79–2.80 * 3.02 3.10	2.80–2.87 * 2.85 3.01

* intramolecular.

**Table 2:** Attribution of peaks/bands present in the FT-IR spectrum of the *ImeTQ* sample to the absorptions of the imepitoin molecule.

Vibration	Wavenumbers, cm^−1^
C=O stretching	1703.4
C=N and aromatic ring stretching	1602.9 1581.8
Aromatic ring stretching	1495.1
Aromatic ring in-plane bending	1281.7 1261.3 1154.1
Combined C-Cl and ring stretching	1113.0
CH out-of-plane bending (2 adjacent H)	819.3
C-Cl stretching	667.0

## Data Availability

The data presented in this study are available on request from the corresponding author.

## Supplementary Materials

The following supporting information can be downloaded at https://www.mdpi.com/article/10.3390/molecules29081724/s1: Figure S1: DSC curves of *ImeTQ* and *ImeGR*; Figure S2: XRPD patterns of *ImeTQ* and *ImeGR*. Figure S3: Raman spectra for ImeTQ (black line) and ImeXyl; Figure S4: H1···O1 distances in polymorph I and polymorph II; Figure S5: Imepitoin polymorph I crystal structure viewed down the (a) *a*-, (b) *b*-, and (c) *c*-axes; Table S1: X-ray Crystallographic data collection and structure refinement for imepitoin polymorph II; Table S2: Comparison of the H···A and C···A (A = N, O, Cl) distances for the two polymorphic forms. Imepitoin polymorph II: imepitoin_refined.cif file. References [24,35,47,48,49] are cited in the supplementary materials.
