# Supplementary material for: New Findings on the Crystal Polymorphism of Imepitoin
Source: Molecules. 2024 Apr 11;29(8):1724. doi: 10.3390/molecules29081724 (PMC11052430; doi:10.3390/molecules29081724)
Supplement: Supplementary file 1 [file molecules-29-01724-s001.zip › molecules-2906834-supplementary.pdf]

Supplementary Material for:

New findings on crystal polymorphism of imepitoin

Giovanna Bruni [1,*], Doretta Capsoni [1], Anna Pellegrini [1], Angela Altomare [2], Mauro Coduri [1], Chiara Ferrara [3], Pietro Galinetto [4], and Renato Molteni [5]

[1] Department of Chemistry, Physical Chemistry Section & C.S.G.I. (Consorzio Interuniversitario per lo Sviluppo dei Sistemi a Grande Interfase), University of Pavia, 27100 Pavia, Italy; giovanna.bruni@unipv.it (G.B.); doretta.capsoni@unipv.it (D.C.); anna.pellegrini01@universitadipavia.it (A.P.); mauro.coduri@unipv.it (M.C.)
[2] Institute of Crystallography – CNR, Via Amendola 122/o, Bari, I-70126, Italy; angela.altomare@ic.cnr.it (A.A.)
[3] Department of Materials Science, University of Milano-Bicocca, via Cozzi 55, 20125 Milano, Italy; chiara.ferrara@unimib.it (C.F.)
[4] Department of Physics, via Bassi 6, 27100 Pavia, Italy; pietro.galinetto@unipv.it P.G.)
[5] A.M.S.A. Anonima Materie Sintetiche Affini S.p.A., Viale Giuseppe Di Vittorio 6, 2100 Como, Italy; r.molteni@amsacomo.it (R.M.)
* Correspondence: giovanna.bruni@unipv.it

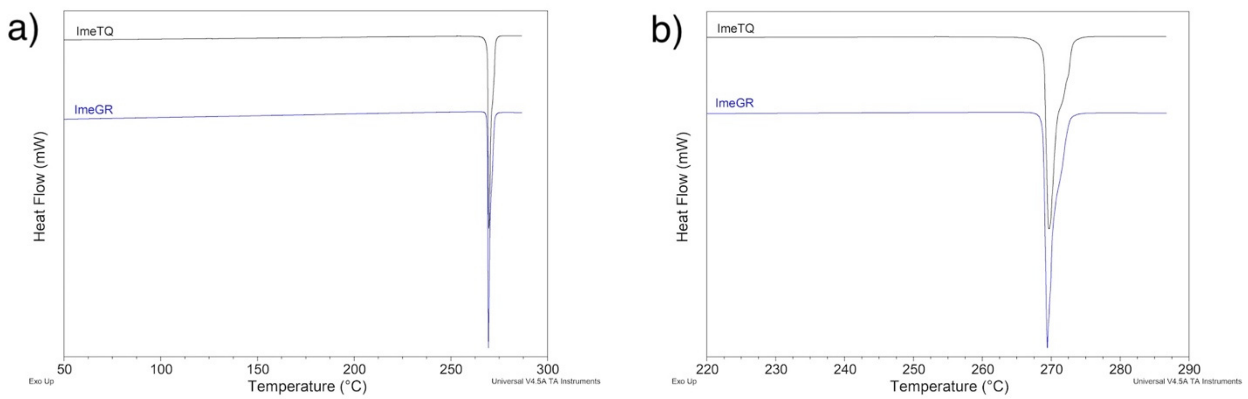

Figure S1- DSC curves of *ImeTQ* and *ImeGR* at 10 K min^{-1}: a) temperature scale from 50 °C to 300 °C; b) the same DSC curves with the temperature scale expanded (from 220 °C to 290 °C).

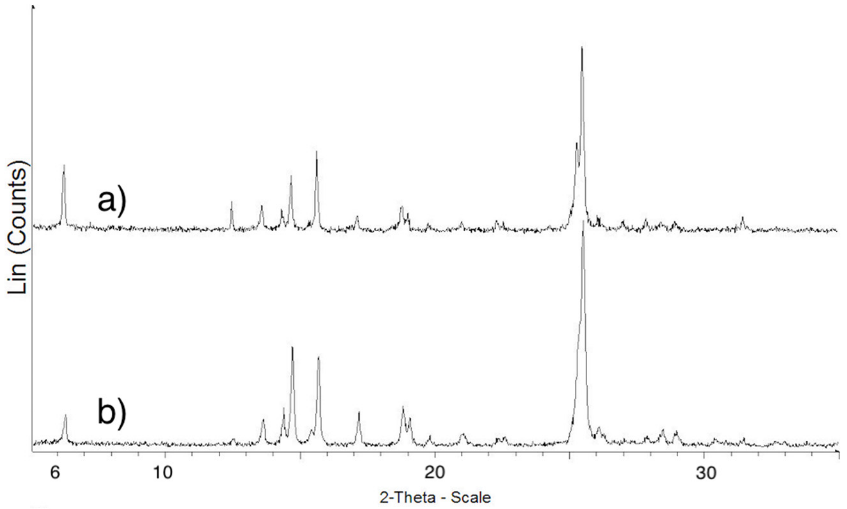

Figure S2 – XRPD patterns of *ImeTQ* (a) and *ImeGR* (b).

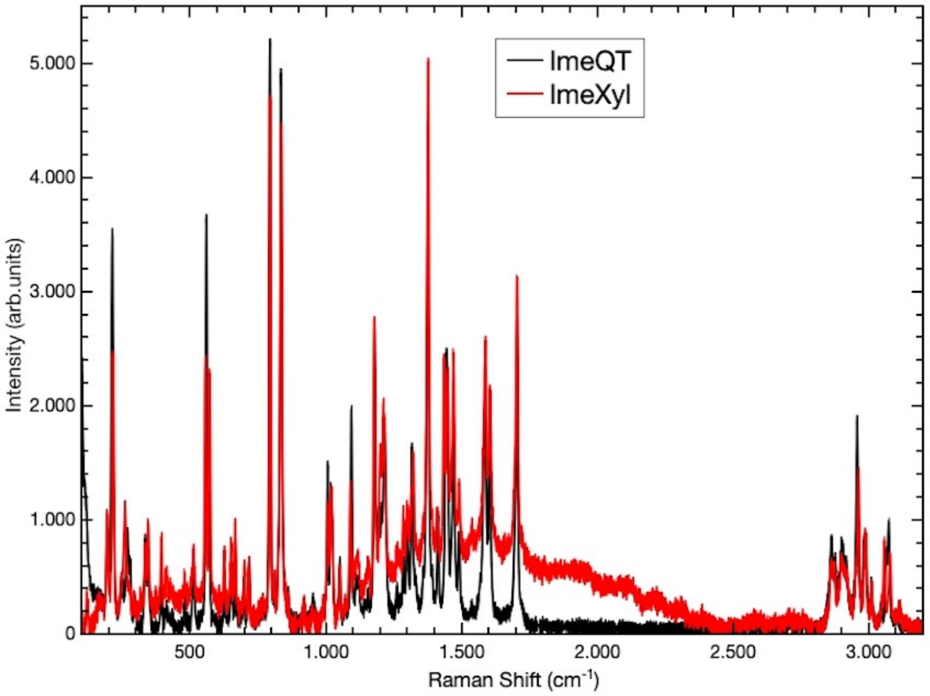

ImeQT
ImeXyl
Intensity (arb.units)
Raman Shift (cm-1)
0
1.000
2.000
3.000
4.000
5.000
500
1.000
1.500
2.000
2.500
3.000

(**a**)

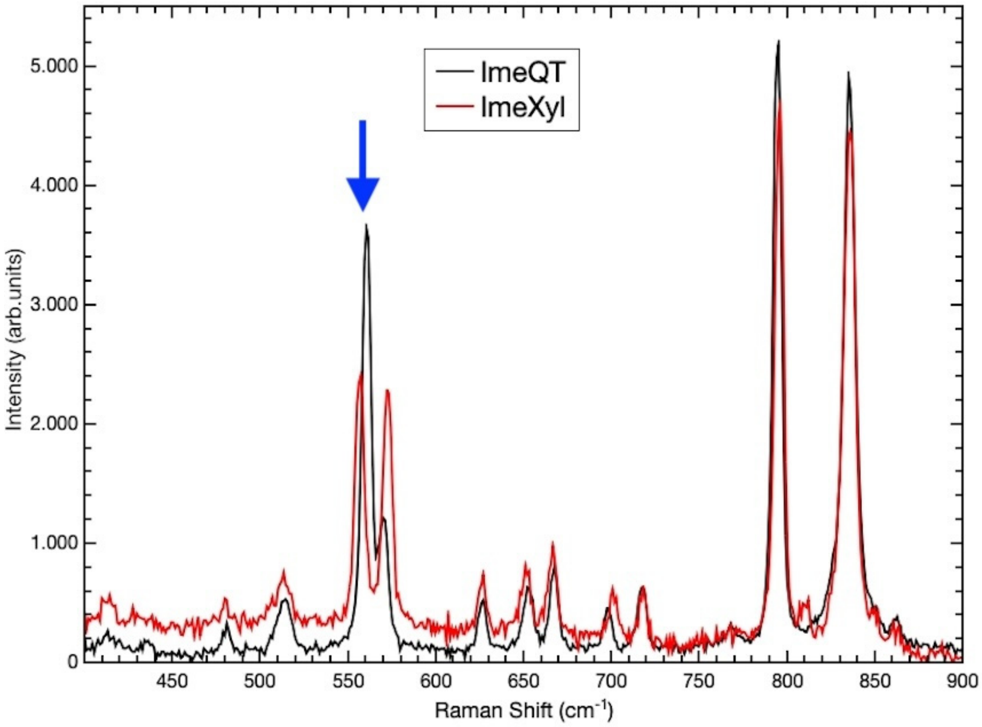

ImeQT
ImeXyl
Intensity (arb.units)
Raman Shift (cm-1)
0
1.000
2.000
3.000
4.000
5.000
450
500
550
600
650
700
750
800
850
900

(**b**)

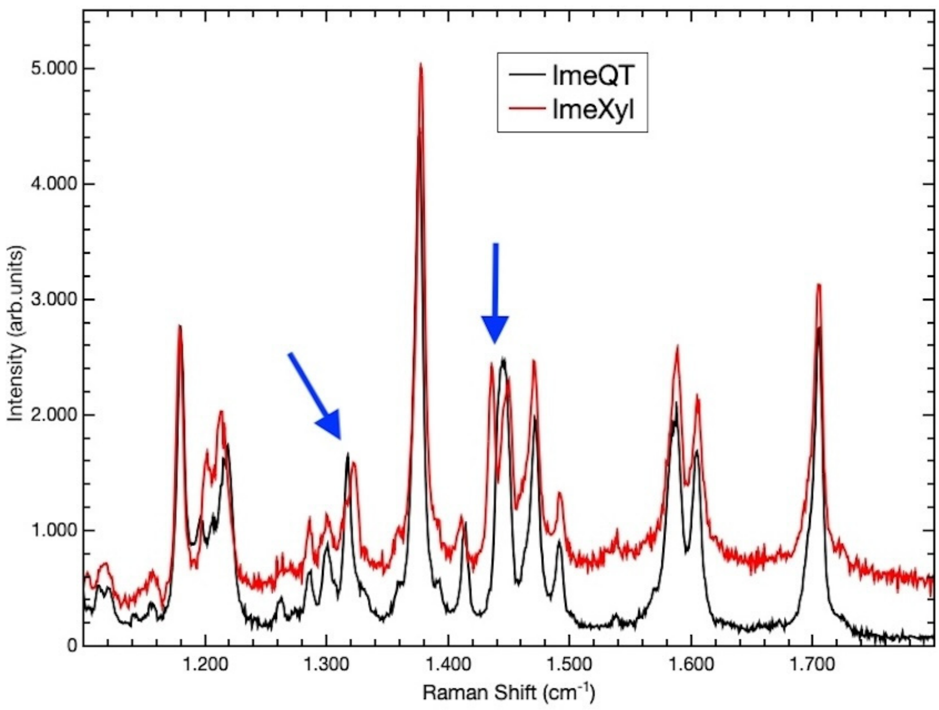

(c)

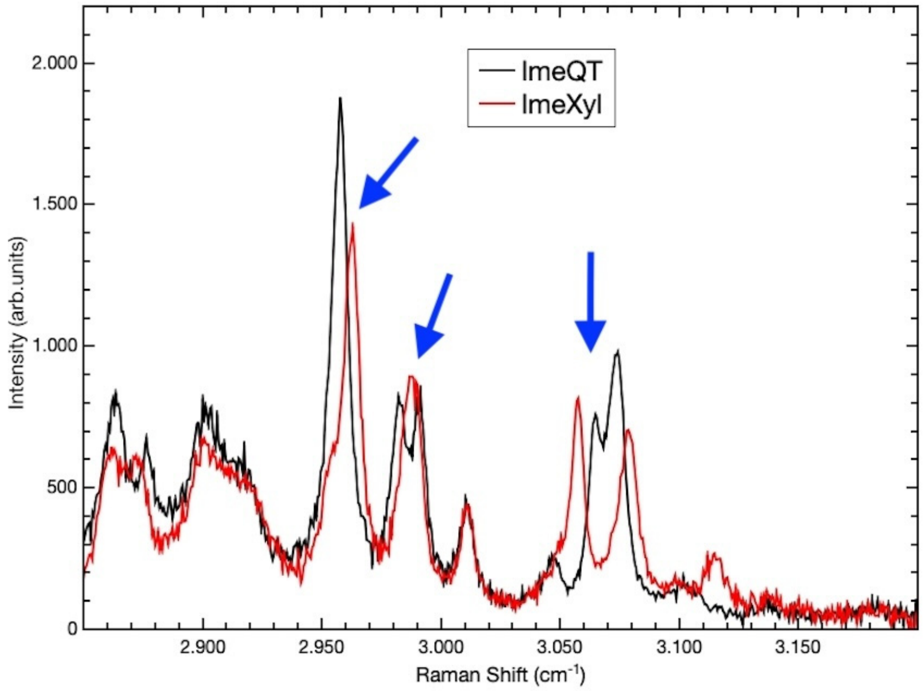

(d)

Figure S3 - a) The whole Raman spectra for *ImeTQ* (black line) and *ImeXyl* (red line). The enlarged spectra in the energy regions; b) between 400 and 900 cm^{-1}, c) 1100-1800 cm^{-1} and d) 2850-3100 cm^{-1}. The arrows indicate the main observed spectral changes.

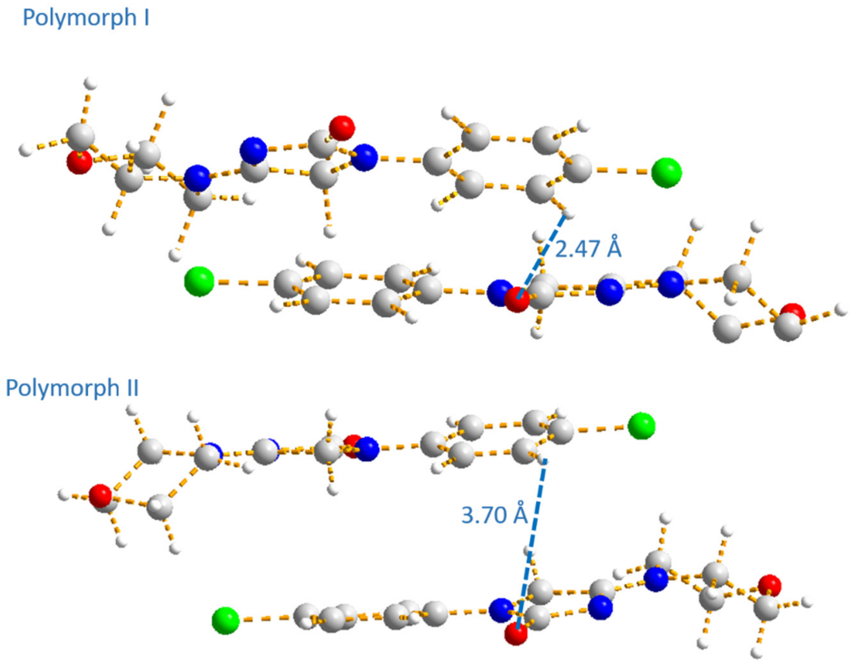

Figure S4 - H1···O1 distances in Polymorph I and Polymorph II.

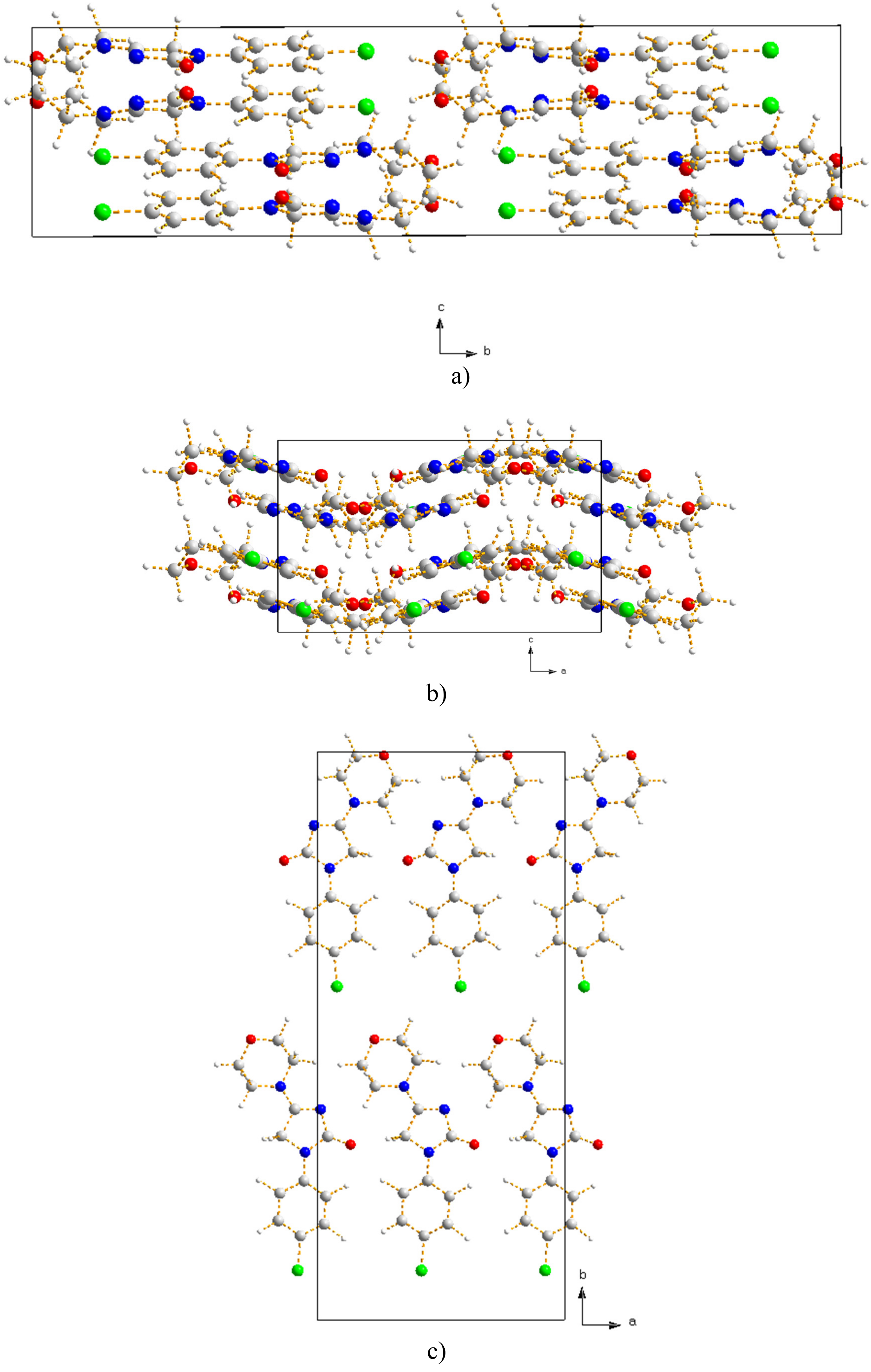

Figure S5 - Imepitoin polymorph I crystal structure viewed down a) *a*-, b) *b*- and c) *c*-axes.

Table S1 - X-ray Crystallographic Data Collection and Structure Refinement for imepitoin polymorph II.

**Crystal data**	**Imepitoin Polymorph II**
Chemical formula	$C_{13}H_{14}ClN_3O_2$
Formula weight (g/mol)	279.72
Crystal system	Monoclinic
Space group	$P2_1/c$
Temperature (K)	298
Cell parameters (Å,°)	a = 14.8687(6)<br>b = 7.2434(2)<br>c = 12.5592(4)<br>β = 107.5586(8)
Volume (Å^3)	1289.61(8)
Z	4
Z′	1
Radiation type	Synchrotron radiation,<br>λ = 0.35418 Å
**Data collection**	
Beamline	ID22@ESRF
Specimen mounting	borosilicate capillary
Data collection mode	transmission
2θ (°)	$2\theta_{min}$ = 0.00<br>$2\theta_{max}$ = 36.00
**Structure solution**	
Methods	Direct methods, simulated annealing (SA)
Parameters (SA)	6+2 DOF, 19 non-hydrogen atoms
Cost function (SA)<br>Building the starting model (SA)<br>Geometry optimization (SA)	R_{wp} = 17.251<br>ACD/ChemSketch [1]<br>MOPAC2016 [2]
**Refinement**	
R_p	5.447
R_{wp}	8.104
R_{exp}	6.246
R_{Bragg}	6.576

χ^2	1.277
No. of data points	9750
No. of reflections	1169
Profile function	Pearson VII
Refinement parameters	
Lattice	4
Positional	57
ADP	3
Profile	10
Background	19
Zero error	1
Restraints	50
H-atom treatment	H-atom parameters constrained
**Programs**	
Indexing	*TOPAS4.2* [3]
Space group determination	EXPO [4]
Structure solution and refinement	EXPO [4]
Model building	Diamond (version3.0)

Table S2 – Comparison of H···A and C···A distances (Å) for the two polymorphic forms. The polymorph I data are taken from table 1 reported by Kaduk et al [5].

	**Polymorph II**		**Polymorph I**	
**H-Bond**	**H···A (Å)**	**C···A (Å)**	**H···A (Å)**	**C···A (Å)**
C8-H4···O1	2.34	3.24	2.13	3.20
C13-H14···O1	2.24*	2.87	2.18	2.90
C1-H1···O1	3.70	3.74	2.47	3.23
C12-H12···O1	2.78	3.71	2.66	3.69
C10-H8···O2	2.70	3.49	2.57	2.48
C11-H11···N3	2.41*	2.83*	2.38*	2.85*
C9-H6···Cl1	3.25	4.24	3.06	4.00

[1] ACD/ChemSketch, Advanced Chemistry Development, Inc.: Toronto, ON, Canada, 2003.

[2] MOPAC2016, Version 18.305L, in: J. J. P. Stewart, Stewart Computational Chemistry, Colorado Springs, CO, USA. http://OpenMOPAC.net/.

[3] *TOPAS4.2*; Bruker: Karlsruhe, Germany, 2009.

[4] Altomare, A.; Cuocci, C.; Giacovazzo, C.; Moliterni, A.; Rizzi, R.; Corriero, N.; Falcicchio, A. EXPO2013: a kit of tools for phasing crystal structures from powder data. *J. Appl. Cryst.* **2013**, *46*, 1231–1235.

[5] Kaduk, J.A.; Gindhart, A.M.; Gates-Rector, S.; Blanton, T.N. Crystal structure of imepitoin, $C_{13}H_{14}ClN_3O_2$. *Powder Diffr.* **2022**, *37*, 206-2010.
